# Laboratory Diagnostics Accuracy for COVID-19 versus Post-COVID-19 Syndrome in Lung Disease Patients with Multimorbidity

**DOI:** 10.3390/jpm14020171

**Published:** 2024-01-31

**Authors:** Daniela Robu Popa, Oana Elena Melinte, Mona-Elisabeta Dobrin, Andrei Tudor Cernomaz, Cristina Grigorescu, Alexandra Floriana Nemes, Doina Adina Todea, Damiana Maria Vulturar, Ionela Alina Grosu-Creangă, Tiberiu Lunguleac, Antigona Carmen Trofor

**Affiliations:** 1 Discipline of Pneumology, III-rd Medical Department, Faculty of Medicine, “Grigore T. Popa” University of Medicine and Pharmacy, 700115 Iasi, Romania; daniela.robu-popa@d.umfiasi.ro (D.R.P.); tudor.cernomaz@umfiasi.ro (A.T.C.); ionela-alina-i-grosu@d.umfiasi.ro (I.A.G.-C.); antigona.trofor@umfiasi.ro (A.C.T.); 2 Clinical Hospital of Pulmonary Diseases, 400012 Iasi, Romania; elisabeta-mona.dobrin@pneumo-iasi.ro; 3 Discipline of the Thoracic Surgery, Faculty of Medicine, “Grigore T. Popa” University of Medicine and Pharmacy, 700115 Iasi, Romania; cristina.grigorescu@umfiasi.ro (C.G.); tiberiu.lunguleac1@umfiasi.ro (T.L.); 4 Doctoral School, Faculty of Medicine, “Titu Maiorescu” University, 031593 Bucharest, Romania; alexandra.nemes@prof.utm.ro; 5 Discipline of Pneumology, Department of Medical Sciences, Faculty of Medicine, “Iuliu Hatieganu” University of Medicine and Pharmacy, 400012 Cluj, Romania; dtodea@umfcluj.ro (D.A.T.); vulturar.damianamaria@elearn.umfcluj.ro (D.M.V.)

**Keywords:** post-COVID-19 syndrome, COVID-19 infection, comorbidities, biological markers, diagnostic accuracy

## Abstract

The laboratory tests and identification of risk factors such as comorbidities are essential in the management, treatment and prognosis of patients with chronic respiratory diseases. Performing rigorous monitoring among patients with post-COVID-19 syndrome and early identification of risk factors associated with poor prognosis are crucial in improving patient outcomes. In the present study, 182 patients diagnosed with COVID-19 and PCI during 2020–2022 were included. A clinical and epidemiological evaluation was performed for each patient. Laboratory tests at admission included complete blood count, Erythrocyte Sedimentation Rate (ESR) and biochemical tests. Receiver operating curve (ROC) and area under the curve (AUC) were calculated to compare the diagnostic performance of each parameter. Regarding comorbidities, arterial hypertension, diabetes mellitus and obesity were the most frequent ones. In the case of chronic lung diseases, asthma and Chronic Obstructive Pulmonary Disease (COPD) were the most frequent. Pleurisy was found especially in patients with PCI Variations in serum LDH values were observed, especially in severe forms of COVID-19 in 2020, with a mean value of 481.44 U/L, compared to patients with PCI, whose mean values (122 U/L) were within the biological range of reference. High neutrophil/lymphocyte ratio (NLR) values quantified in this study were especially associated with moderate and severe forms of COVID-19 and also PCI. The Spearman correlation coefficient was determined to measure the correlations between the clinical parameters of all investigated subjects. A value of *p* < 0.05 was considered statistically significant. The statistical results indicated that serum lactate dehydrogenase (LDH), glucose and C-reactive protein (CRP) are sensitive markers with a diagnostic role in COVID-19, and lymphocyte (Ly) count, CRP, ESR and glucose were evidenced to be target markers in PCI. LDH values were observed to be statistically significant (*p* < 0.005) in patients with COVID-19 and obesity evaluated in 2021, while Ly count was statistically significant (*p* = 0.05) in patients with PCI and arterial hypertension. Regarding comorbidities, it has been observed that obesity, arterial hypertension and cardiovascular diseases represent risk factors in COVID-19/PCI, associated especially with the severe forms of the disease.

## 1. Introduction

The etiological agent of COVID-19 (Coronavirus Disease 2019) with a pandemic evolution is the SARS-CoV-2 virus (Severe Acute Respiratory Syndrome Coronavirus 2), with an impressive worldwide epidemiology, with over 770 million diagnosed cases and over 6.9 million deaths. In Romania, almost 3.5 million cases have been registered, of which 68,568 are deaths; these data underline the importance that must be given to this condition, both in the acute stage and later in the follow-up phase [1]. Considering the specific heterogeneity of COVID-19, the clinical appearance can vary considerably, from mild forms, with discrete or even absent symptoms (aspects observed in most patients), to forms with a rapid unfavorable evolution, with the development of cytokine storm, acute hypoxemic respiratory failure and multiple organ dysfunction syndrome (MODS); all these aspects are more prevalent among vulnerable patients [2,3,4,5]. Patients with severe forms of COVID-19 can develop acute respiratory distress syndrome (ARDS), with increased rates of morbidity and mortality [4]. According to World Health Organization, the development of post-COVID-19 syndrome or Long COVID-19 can occur in patients with a history of probable or confirmed SARS-CoV-2 infection, which typically develops beyond 3 months after the acute viral episode, persists for at least 2 months and cannot be explained by other diagnoses [6,7,8]. In addition, the latest studies in the field showed an increasing addressability even after 6–12 months after the acute viral episode, especially among patients who have developed moderate and severe forms, which makes a rigorous follow-up in order to minimize long-term effects imperative [9,10]. 

Studies have showed the existence of hematological and biochemical markers useful in the diagnosis, management and prognosis of COVID-19, as well as for Long COVID, such as neutrophilia, lymphopenia, thrombocytopenia, increased neutrophil/lymphocyte ratio (NLR) and increased inflammatory markers, such as CRP (C-Reactive Protein), ESR (Erythrocyte Sedimentation Rate) and LDH (lactate dehydrogenase) [2].

In the mild forms of COVID-19, one of the most common hematological changes encountered is lymphopenia. On the other hand, patients who developed moderate–severe forms of COVID-19 presented other laboratory changes associated with lymphopenia, such as neutrophilia, elevated D-dimer and reduced fibrinogen levels [5].

Patients with critical forms of COVID-19, with an important inflammatory status, have neutrophilia, lymphopenia and an increased NLR; these parameters are markers for the imminent development of an ARDS [5,7,11,12].

The neutrophil/lymphocyte ratio is directly proportional to the risk of morbidity and mortality. Increased NLR may be the consequence of neutrophilia associated with a bacterial infection in severely immunocompromised patients and can be used in clinical practice as an important early prognostic factor of unfavorable disease evolution. On the other hand, increased NLR was also observed among patients with COVID-19 without associated bacterial superinfection [7,13,14,15]. In the context of the cytokine storm and the systemic inflammatory status, patients will show persistently elevated levels of inflammatory markers, such as erythrocyte sedimentation rate, CRP, pro-calcitonin, LDH and ferritin [2,5]. Patients who have developed moderate–severe forms of COVID-19 showed an increased level of CRP from the early stages of the disease, which can be correlated with the magnitude of the imaging changes [2,7,16]. It seems that the number of platelets is inversely proportional to the severity of the disease; this is also mentioned regarding LDH levels. When these values are higher, patient prognoses are more likely to be unfavorable [2,7,17].

Performing a rigorous follow-up among patients with post-COVID-19 syndrome and early identification of the risk factors associated with poor prognosis are crucial in improving patient outcomes. This study aims to evaluate the contribution of some sensitive laboratory markers regarding the severity and prognosis of COVID-19; this study also investigates their utility in the early diagnosis and management of the disease, both in the acute phase and later, in the Long COVID-19 stage. Until now, there have been few comparative studies of COVID-19/post-COVID-19 syndrome that have highlighted the statistically significant differences in laboratory parameters that have an important role in the accuracy of the diagnosis and in prognosis.

## 2. Materials and Methods

### 2.1. Study Design and Participants

This analytical, retrospective and observational study recruited adult patients diagnosed with COVID-19 and PCI between 2020 and 2022. The patients were diagnosed at the Clinical Hospital of Pneumology, Iasi, Romania. The diagnosis of COVID-19/PCI was made following the guidelines of the World Health Organization [18]. Exclusion criteria included pregnant women, children and patients with hemolyzed biological samples. Patients were selected from the hospital’s electronic system, comprising patients diagnosed with SARS-CoV−2 infection during the Delta and Omicron waves. The study was approved by the hospital’s ethics committee and the “Grigore T. Popa” University of Medicine and Pharmacy in Iasi. The clinical status of patients was monitored from hospital admission to discharge.

### 2.2. Quantification of Biomarkers and Analytical Quality Control

Blood samples were collected on admission for all study participants. Taking all aseptic precautions, about 7 mL of blood was drawn by venipuncture from a peripheral vein with a disposable syringe, then collected in a clean dry glass tube (clot activator tube) that allowed it to stand for 10–15 min at room temperature for the retraction of the clot. This was centrifuged at 4000 rpm for 10 min to separate the blood serum. 

All tests were performed in the biochemical laboratory department, following standard procedures for clinical biochemistry purposes. The biological parameters measured were the following: CRP, glucose and LDH. Biochemical assay serum parameters were measured using a Cobas Integra 400 plus (Roche Diagnostics GmbH, Mannheim, Germany) biochemical autoanalyzer. The quality assurance of the results was reported by performing the daily internal quality control and using control sera for normal and pathological levels. The Biochemistry Laboratory participates annually in the external quality control scheme and reported a z-score <1.5 for all investigated parameters. Hematological assay platelet count (PLT), neutrophil percent (N%) and lymphocyte percent (L%) were assessed using Hematology Analyzer Mindray Sysmex_XN1000.1 (Sysmex Corporation, Kobe, Japan).

### 2.3. Statistical Analysis

All statistical results were obtained using the Statistica 10 package (StatSoft USA) and R Statistic Software program (R verwsion 4.3.2). Descriptive statistics were performed using the Microsoft Excel program and for each biochemical and hematological parameter determined in patient’s average, standard deviation and range. The normality of the results was tested using the Kolmogorov–Smirnov test. The Spearman correlation coefficient was determined to measure the correlations between clinical parameters of all investigated subjects. A *p*-value of <0.05 was considered statistically significant. Because the analyzed variables are not normally distributed, for the comparisons made between them depending on the lot of the patient evaluated (patients with COVID-19/PCI investigated in 2020, 2021 and 2022), we used the nonparametric Wilcoxon rank sum and Mann–Whitney tests. The receiver operating curve (ROC) and area under the curve (AUC) were calculated to compare the diagnostic performance of each parameter. A value of *p* < 0.05 was considered statistically significant.

## 3. Results

Demographic data, clinical examination, biochemical and hematological markers and hospitalization periods were extracted from the hospital’s database and processed statistically. Based on the severity of COVID-19, all patients were classified into three groups: those with moderate, moderate/severe or severe forms of SARS-CoV−2 infection [15].

Patients diagnosed with COVID-19 (n = 79) came from both urban (40) and rural (39) areas, with an average age between 57.3 and 68.10, and presented various comorbidities, such as arterial hypertension, diabetes mellitus, obesity and respiratory conditions, such as Chronic Obstructive Pulmonary Disease (COPD) and bronchial asthma being the most prevalent (Table 1). Patients with PCI (n = 103) also originated from urban (n = 53) and rural (n = 50) areas, with an average age between 60.76 and 63.85 years, and exhibited comorbidities, primarily arterial hypertension, obesity and diabetes mellitus, as well as non-specific pleurisy and tuberculosis (Table 1).

For each patient, a clinical and epidemiological evaluation was performed. Laboratory tests at admission included a complete blood count, blood chemistry and the inflammatory biomarker named C-reactive protein (CRP). The group of patients diagnosed with COVID-19 between 2020 and 2022 predominantly exhibited a moderate form of the disease.

We conducted an evaluation of laboratory parameters based on the severity of the diagnosis to identify key paraclinical changes with a diagnostic role in COVID-19/PCI. Evident abnormalities in laboratory analyses observed in our study included an increase in the number of neutrophils, a decrease in the number of lymphocytes, and an increase in ESR, especially in moderate and severe forms of COVID-19. Lymphopenia was accentuated, particularly in moderate and severe forms of COVID-19, for patients diagnosed in the year 2020 (Table 2). Additionally, the number of lymphocytes was much lower in patients with COVID-19 compared to PCI, evaluated in the years 2021 and 2022 (Table 3 and Table 4).

### Profile of the Biochemical and Hematological Results in COVID-19/PCI

In this retrospective study, 182 patients diagnosed with COVID-19 during the period 2020–2022 and patients diagnosed with PCI during the period 2021–2022 were included. The most common clinical manifestations in COVID-19 patients at admission were cough, fever, difficulty in breathing and rhinorrhea. Among patients with COVID-19, 46% had arterial hypertension, 16% had obesity and 25% had diabetes mellitus. In the case of PCI patients, 35% had arterial hypertension, 11% had diabetes mellitus and 13% had obesity. Sixteen of the patients diagnosed with COVID-19 in the period 2020–2022 returned to the healthcare facility where they were clinically and paraclinical evaluated and they were subsequently diagnosed with PCI. In the case of chronic lung diseases, asthma and COPD were the most common. Of the patients, 10% with COVID-19 had COPD, and in the case of patients with PCI, 11% had COPD. Of the patients, 6% with COVID-19 had a diagnosis of asthma, and among those with PCI, 9% had asthma. Pleurisy was found in 10% of patients with PCI investigated in 2021 and in 27% of patients with PCI investigated in 2022. Bronchopulmonary neoplasm was present in 4% and 8% of patients with PCI evaluated in 2021 and in 2022, respectively. Heart diseases were present especially in patients with COVID-19 in the years 2021/2022, and a small number of the investigated patients had hypothyroidism and pulmonary tuberculosis. (Table 1)

Table 1 presents the demographic and clinical characteristics of the patients included in the study. At admission, the relevant abnormalities in blood analyses were noted, particularly regarding hematological parameters and the profile of inflammatory markers. Descriptive statistics results were expressed in terms of mean, standard deviation and range (minimum value–maximum value). In the case of patients diagnosed with COVID-19 in the year 2020, descriptive statistics indicated significant changes in the lymphocyte and neutrophil counts, as well as elevated values for CRP and LDH in severe forms of SARS-CoV−2 infection (Table 2). Variations in serum LDH values were observed, especially in severe forms of COVID-19 in the year 2020, with an average value of 481.44 U/L, compared to PCI patients, whose mean values fell within the biological reference interval (BRI), specifically 122 U/L.

**Figure 1:**
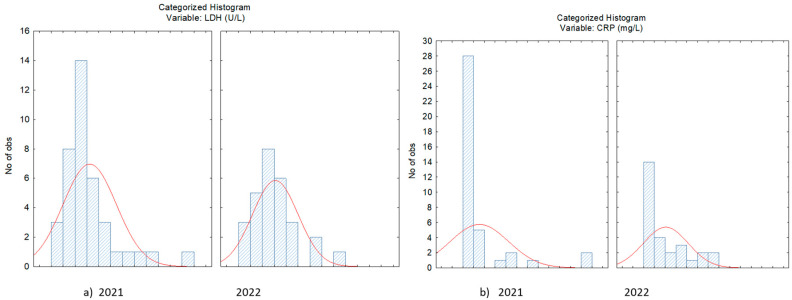
Distribution of LDH (**a**) and CRP (**b**) concentration in case of patients with SARS-CoV−2 infection evaluated in 2021 and 2022.

Hemocytopenia was frequent, including lymphopenia and thrombocytopenia. Hematological and biochemical parameters were compared between groups of patients with COVID-19 and PCI, investigated in the years 2020, 2021 and 2022. The comparative study of biological variables showed elevated values for CRP in COVID-19 versus PCI (28.90/8.72 mg/L; 82.22/58.48 mg/L; 131.30/58.48 mg/L) and LDH (554.56/284.12) for patients diagnosed in the year 2021. High values for both LDH and CRP were also observed in the year 2021, compared to 2022, when the Delta and Omicron forms of SARS-CoV−2 infection were identified (Figure 1a,b).

The values of lymphocyte counts were significantly reduced in patients with COVID-19 compared to PCI, with a notable difference (*p* < 0.05) observed between the values obtained for the patient group diagnosed in 2021 compared to those diagnosed in 2022. Platelet counts were altered, especially for patients diagnosed with COVID-19 in the years 2020–2021 when the Delta variant of the SARS-CoV−2 virus was identified in Romania. Erythrocyte sedimentation rate (ESR) values were generally higher, especially for moderate forms of COVID-19, but also in PCI (Table 3).

A useful biomarker of systematic inflammation is NLR. This ratio is frequently used in bacterial infections to predict the status of patients, especially those with pneumonia. NLR provides essential information about the prognosis of patients with various pathologies, from neoplastic to other inflammatory diseases, such as acute coronary syndrome, intracerebral hemorrhage, polymyositis and dermatomyositis [13]. Significantly higher values of this ratio were observed in severe and moderate forms of COVID-19/PCI (N/L = 5.18/6.410; N/L = 5.20/7.17). 

**Figure 2:**
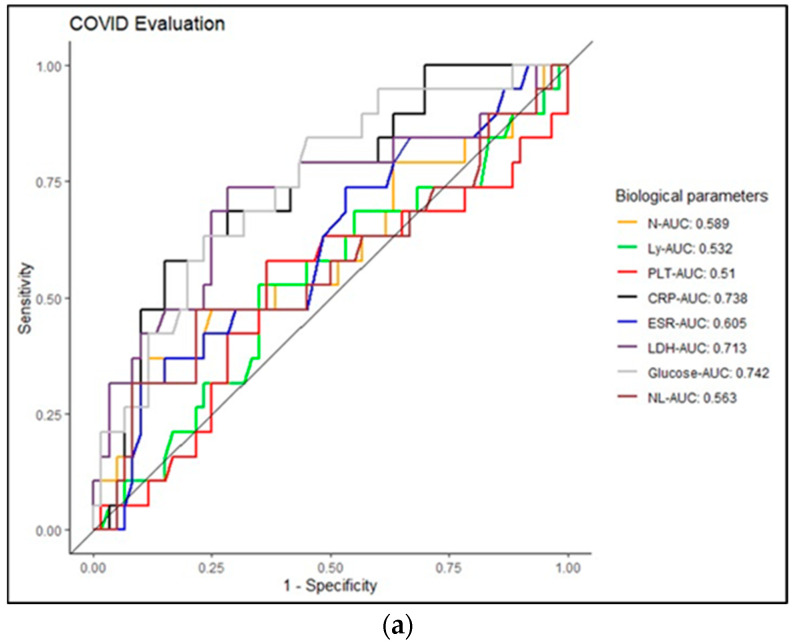

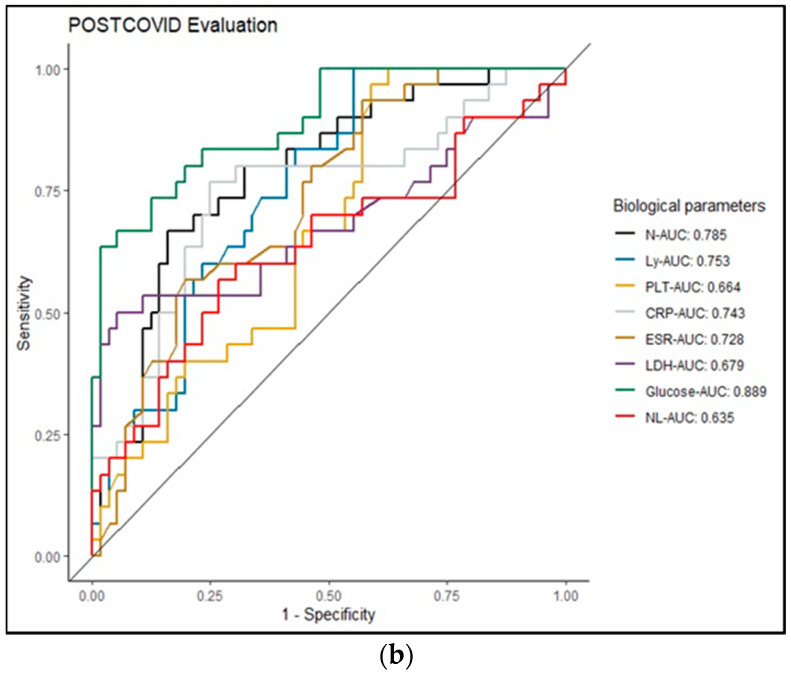
(**a**) Representation of the ROC curve in COVID-19 patients. (**b**) Representation of the ROC curve in case of patients with PCI.

Serum glucose concentration values were higher than the biological reference interval (BRI), especially in patients with moderate and severe forms of COVID-19 (Table 2, Table 3 and Table 4). In patients with PCI, moderate and severe forms of COVID-19/PCI were associated with average values of serum glucose within the BRI (Table 3 and Table 4). To study the specificity and sensitivity of the biological variables, we used the ROC curve (Figure 2a), which allowed us to specify the area under the curve of each biomarker, called the AUC (area under the curve). The ROC curve plots the true positive rate (sensitivity) against the false positive rate (1—specificity) for different thresholds of the test or model. As described in Figure 2a, the calculated AUCs for patients with COVID-19 are presented as follows: CRP—0.743; glucose—0.742; LDH—0.713; ESR—0.605; N—0.589; Ly—0.532; N/L—0.563; PLT—0.51 (Figure 2a). In the case of PCI, the calculated AUC indicated the following: glucose—0.889; N—0.785; LY—0.753; CRP—0.743; ESR—0.728; LDH—0.679; N/L—0.635; PLT—0.664 (Figure 2b).

**Figure 3:**
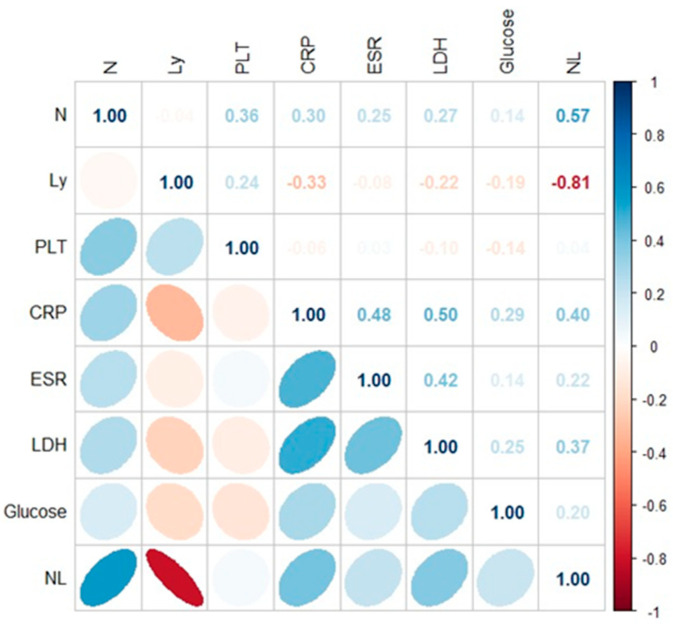
Application of the Spearman correlation to biological variable in COVID-19 patients.

**Figure 4:**
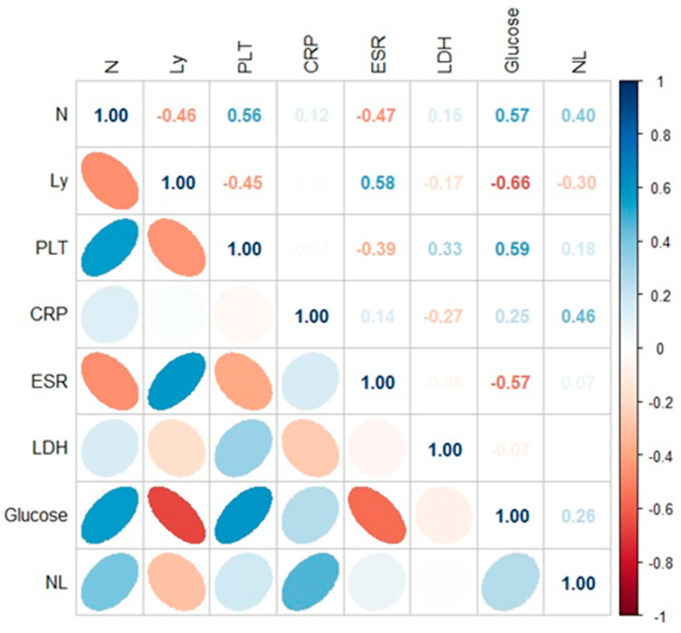
Application of the Spearman correlation to biological variable in PCI patients.

Spearman’s rank correlation coefficient was used to measure the strength of the correlations between biochemical and hematological parameters corresponding to the patients with COVID-19 (Figure 3) and PCI infection (Figure 4). A *p*-value less than 0.05 was considered to indicate statistical significance. Significant positive correlations were observed between biochemical and hematological parameters in case of COVID-19 patients (CRP × N/L (r = 0.40; *p* < 0.05), CRP × LDH (r = 0.50; *p* < 0.05); ESR × LDH (r = 0.42; *p* < 0.05); LDH × N/L (r = 0.37; *p* < 0.05). In the case of patients with PCI, the following correlations were identified: Ly × glucose (r = 0.66; *p* < 0.05); N × glucose (r = 0.57; *p* < 0.05); PLT × glucose (r = 0.59; *p* < 0.05); CRP × N/L (r = 0.46; *p* < 0.05) (Figure 3 and Figure 4).

Differences in laboratory biomarkers according to the severity of COVID-19/PCI infection was tested with the Mann–Whitney U test. The Mann–Whitney U test is a non-parametric statistical test used to determine whether there is a significant difference between two independent unpaired groups. The results highlighted that the patients diagnosed with COVID-19 in 2020 and 2021 have a different biological profile. The lymphocyte and neutrophil count, CRP, LDH, age and hospitalization time differed statistically (*p* < 0.005; *p* < 0.0005) in all the investigated groups (Table 5). Furthermore, the statistical results presented in the Mann–Whitney U test show a significant statistical difference for the number of lymphocytes in the COVID-19/PCI study group in 2021, as well as in the COVID-19/PCI group in 2022 (Table 5).

**Figure 5:**
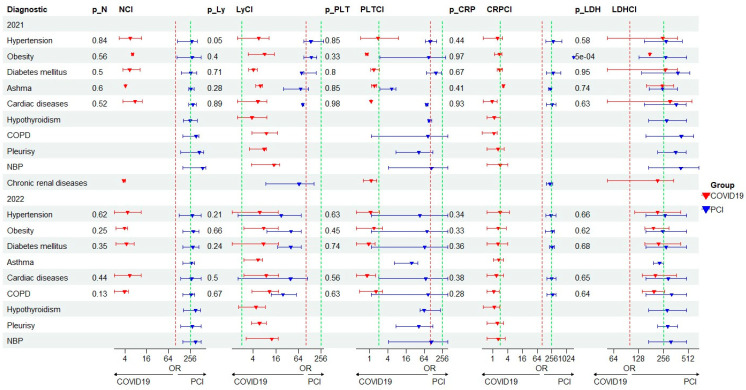
Forest plot for biological variable in COVID-19/PCI patients with multimorbidity. N—neutrophils; Ly—lymphocytes; PLT—platelets; CRP—C-reactive protein; LDH—lactate dehydrogenase.

Regarding comorbidities, approximately 42% of the investigated patients have at least one comorbidity, with the most common being arterial hypertension both in patients with COVID-19 and in those with PCI. Obesity was the most common in patients with PCI and diabetes mellitus was predominant in patients with COVID-19 (Table 1). A forest plot was created to describe all the biological parameters with high importance in COVID-19/PCI in the case of associated comorbidities (Figure 5). It was observed that LDH values have statistical significance (*p* < 0.005) in the case of patients with COVID-19 and obesity evaluated in 2021, while the number of Ly shows a statistical significance (*p* = 0.05) in patients with PCI and hypertension, investigated in the year 2021 (Figure 5).

## 4. Discussion

Many data from the literature illustrate lymphopenia in a significant number of patients, attributed to immune mechanisms mediated by proinflammatory mediators or even the exudation process of circulating lymphocytes in the lung tissues affected by inflammation. It was observed that a sustained decrease in the number of lymphocytes in peripheral blood is an early indicator of patients diagnosed with severe/critical forms of COVID-19 [19,20]. Similar studies have shown that a low lymphocyte count is a negative prognostic factor regarding COVID-19 and can also serve as a sensitive indicator of the severity of the disease [21,22,23].

Studies have also shown that patients with severe evolution of COVID-19 exhibit an association of lymphopenia with neutrophilia, as well as elevated values of LDH and CRP. [11,19]. 

Elevated concentrations of CRP were directly correlated with the level of inflammation and the severity of the disease. Therefore, CRP is an important biomarker in the diagnosis and assessment of the severity of infectious diseases [24]. Therefore, it is considered that the values for CRP and LDH were much higher in the COVID-19 cohorts from the years 2020 and 2021 because patients developed the Delta variant of the SARS-CoV−2 virus. In the year 2022, the Omicron variant prevailed, which is much more contagious than Delta but with a lower risk of hospitalization. It seems that patients infected with the Omicron variant have fewer comorbidities and a lower risk of hospitalization compared to those infected with the Alpha, Gamma, and Delta variants. Regarding the Delta and Omicron variants, a study conducted by Kahn et al. stated that the risk of severe disease and hospitalization from Omicron was markedly lower among vaccinated and unvaccinated cases but remained high among patients with two or more comorbidities and among elderly people [25].

The high values of NLR quantified in this study were particularly associated with moderate and severe forms of COVID-19/PCI. Regarding COVID-19 pathology, studies have shown a significant increase in NLR in severe forms [19]. NLR has been proposed as a quick and simple tool for screening vulnerable patients for respiratory conditions [19]. The results of a study showed that patients with an NLR value greater than 6.5 had severe forms of COVID-19, while NLR values of 9 were associated with increased likelihood of mortality [19]. 

It is important to compare clinical parameters in COVID-19/PCI to estimate key biomarkers in the diagnosis and monitoring of the disease. The predictive threshold values for severity were obtained using ROC curves in our study. Thus, CRP, LDH and glucose can be considered important predictors in evaluating patients with COVID-19 (AUC > 0.7) (Figure 2). In the case of PCI, neutrophils, LDH, CRP and glucose can be considered important biological predictors (AUC > 0.7) (Figure 2b) in diagnosing patients. Many data in the literature have included age, male gender and comorbidities as major factors in the development of severe forms of COVID-19. Generally, individuals over the age of 65 and patients with multiple comorbidities have an increased risk of developing severe forms of COVID-19 and PCI [2]. The early identification of the risk factors associated with the development of the post-COVID-19 syndrome is essential in the management of these categories of patients. According to a systematic literature review and meta-analysis published by Notarte et al., which included a total of 12 articles with 677,045 COVID-19 patients, the development of Long COVID-19 can be associated with the existence of prior medical comorbidities, especially pulmonary diseases, diabetes mellitus, obesity and organ transplantation. At the same time, older age, female sex and pre-existing medical conditions can be predictors of long COVID-19 symptoms [26].

In this study, the most common comorbidities encountered in patients with COVID-19 and PCI were hypertension, diabetes mellitus, obesity and cardiac conditions. Patients with diabetes mellitus can develop more severe forms of COVID-19, with the development of severe complications, such as acute respiratory distress syndrome (ARDS) that require invasive ventilation and referral to the intensive care unit (ICU), compared to patients without diabetes mellitus [27]. Also, patients with diabetes mellitus can face a prolonged and complicated recovery after the acute phase of COVID-19 [28]. Regarding the severity and progression of the COVID-19 disease, studies have shown that they are directly proportional to the number and severity of associated comorbidities. Another systematic review and meta-analysis, which included 187 studies describing 77,013 patients, stated the most prevalent comorbidities being hypertension in mild, moderate, severe and critical forms of COVID-19, followed by diabetes mellitus and cardiovascular diseases. It seems that patients with neurological and autoimmune diseases present the highest risk of unfavorable progression of COVID-19, with intensive care unit (ICU) admission and increased mortality rate [29]. The results of the present study have shown that patients with PCI have multiple comorbidities, and obesity and cardiac conditions represent a significant percentage among them compared to patients with COVID-19. Additionally, it was observed that 41% of the patients with PCI investigated in the year 2022 develop a severe form of the disease and require a greater number of days of hospitalization (Table 1).

Patients with obesity and lipid metabolism disorders have an increased risk for development of Long COVID-19, with increased number and also persistence of symptoms [30]. Studies have shown that the prognosis of patients and the risk of developing severe forms of COVID-19 is decisively influenced by the associated comorbidities (cardiovascular, cerebrovascular and metabolic diseases) and by admission to the intensive care unit, mortality being much higher in the latter group [31]. 

The present study also points to the modification of inflammatory markers in COVID-19/PCI, as well as to the high values of the average concentrations for serum CRP and LDH in patients with chronic respiratory diseases, especially asthma and COPD (Figure 5, Table S1 Supplementary Materials). It was observed that the combination of COPD and Long COVID-19 can lead to exacerbated respiratory symptoms and increased risk of severe complications. If individuals with COPD develop Long COVID-19, they may experience persistent respiratory symptoms, such as shortness of breath, persistent cough, chest pain and fatigue. Considering the systemic inflammatory status and COPD comorbidity, the morbidity and mortality rates associated with COVID-19 and COPD are much higher, and the evolution of these category of patients can be unpredictable, with frequent exacerbations and accelerated decline of lung function [15]. It is essential for such individuals to consult healthcare professionals to assess their condition, manage their symptoms, and create an appropriate treatment plan. For individuals with pre-existing asthma, a SARS-CoV-2 infection can potentially trigger asthma exacerbations or worsening of symptoms. This is because the virus primarily affects the respiratory system, leading to increased inflammation and narrowing of the airways in individuals with asthma [32].

Patients with COVID-19 and asthma experience longer symptom duration, increased inhaler use and worsening asthma management [33].

Patients with PCI with pleurisy presented an important inflammatory syndrome, with high values of the average concentration of CRP and with an average of LDH above the reference interval. This aspect was highlighted in other studies, such as the one by Petr Jakubec and colleagues, in which there were observed more frequent pleural effusions (21.1%) in the group with infectious causes of admission for Long COVID-19, compared to the group with other causes [34]. Further studies are needed to assist clinicians in the early assessment and monitoring of patients with post-COVID-19 syndrome in order to diagnose the complications that may occur and to prevent long-term sequelae that may have a decisive unfavorable impact in patient evolution.

## 5. Study Limitations

The present study has some limitations. First of all, there were few patients diagnosed with post-COVID-19 infection who returned to the hospital unit after the acute phase of the disease. Secondly, the comorbidities were heterogeneous with few cases of chronic lung diseases. Thus, no pertinent conclusion could be drawn regarding the variability of laboratory markers in the case of chronic lung diseases.

## 6. Conclusions

From a statistical point of view, sensitive markers with a diagnostic role in COVID-19 (LDH, Glucose, CRP) and in PCI (Ly count, CRP, ESR and glucose) were highlighted. In addition, serum LDH levels were higher in the acute phase of SARS-CoV-2 infection (2020/2021, delta variant) compared to PCI, where the values approached the Biological Reference Interval. Results pointed out that the patients diagnosed with COVID-19 in 2020 and 2021 have different biological profiles. The lymphocyte and neutrophil count, CRP, LDH, age and hospitalization time differed statistically significantly (*p* < 0.005; *p* < 0.0005) in all the investigated groups. The diagnostic accuracy was supported by the specificity and the sensitivity of the biological biomarkers by ROC curves. Regarding comorbidities, it has been observed that obesity, arterial hypertension and cardiovascular diseases represent risk factors in COVID-19/PCI, associated especially with the severe forms of the disease.

## Figures and Tables

**Table 1:** Description of demographic characteristics and the clinical data of COVID-19/PCI patients hospitalized in Clinical Hospital of Pulmonary diseases, Iasi, Romania.

Characteristics of the Patients	COVID-19/PCI		
	2020	2021	2022
*Age (mean ± stdev)*	57.3 ± 13.37	61.37 ± 14.80/60.76 ± 17.93)	68.10 ± 16.06/63.85 ± 15.12
Man	22 (61%)	16 (66%)/39 (75%)	10 (53%)/29 (57%)
Woman	16 (39%)	8 (34%)/13 (25%)	9 (47%)/22 (43%)
Time for hospitalization	19.5 ± 6.03	13.33 ± 5.46/12.17 ± 7.19	9.52 ± 2.98/9.10 ± 8.82
*Demographic area*			
Area (urban/rural)	20 (56%)/16 (44%)	(10 (42%)/14 (58%)/(24 (46%)/28 (54%)	(10/9)/(29/22)
*COVID-19 forms*	*(n = 36)*	*(n = 24)/(n = 52)*	n = 19/n = 51
Mild	10 (28%)	2 (8%)/6 (11%)	6 (32%)/9 (18%)
Moderate	21 (58%)	13 (54%)/25 (48%)	10 (52%)/31 (60%)
Severe	5 (14%)	9 (38%)/21 (41%)	3 (16%)/11 (22%)
*Comorbidities*			
Hypertension	12 (33%)	13 (54%)/20 (38%)	11 (58%)/16 (31%)
Obesity	2 (5%)	2 (8%)/6 (12%)	2 (10%)/7 (14%)
Diabetes mellitus	8 (22%)	8 (33%)/4 (8%)	4 (21%)/7 (14%)
Asthma	2 (5%)	2 (8%)/6 (12%)	1/ (5%)/3 (6%)
Chronic Kidney diseases	NA	3 (12%)/1 (2%)	0/2/(4%)
Cardiac disease	5 (14%)	7 (29%)/3 (6%)	7 (37%)/4 (8%)
COPD	NA	2 (8%)/3 (6%)	6 (32%)/8 (16%)
Tuberculosis	NA	NA	2 (10%)/2 (4%)
Pulmonary hypertension	NA	NA	0/2 (4%)
Pleurisy	NA	0/5 (10%)	0/14 (27%)
Bronchopulmonary neoplasm	NA	0/2 (4%)	0/4 (8%)
Hypothyroidism	1 (2%)	1 (4%)/6 (12%)	1 (5%)/0

NA = Not applicable.

**Table 2:** Laboratory biomarkers according to the severity of COVID-19 (year 2020).

Year 2020	Mean	Stdev	Range	Mean	Stdev	Range	Mean	Stdev	Range	*p* Value
(n = 36)	Mild Form			Moderate Form			Severe Form			
Neutrophils (×10^3^/µL)	3.10	1.09	1.21–4.75	4.12	2.34	1.21–4.75	3.20	1.24	2.03–5.11	1.00
Ly(×10^3^/µL)	3.52	7.45	0.49–24.7	1.61	0.79	0.49–24.70	1.17	0.87	0.35–2.17	0.59
PLT (×10^3^/µL)	257.30	62.29	162–345	217.38	87.78	162–345	175.40	31.63	138–214	0.01
CRP (mg/L)	26.5	33.8	0.2–94.9	110.6	36.61	0.2–94.9	82.5	60.1	13–160.3	0.03
ESR (mm/1 h)	37.10	34.88	6–120	48.67	39.41	6–120	42.80	32.13	34.8–81.00	0.67
LDH (U/L)	261.78	156.90	172.1–684.3	242.97	72.12	172.1–684.3	481.44	246.99	152.6–744.10	0.20
Glucose (mg/dL)	104.39	19.15	77.7–132	111.06	27.50	77.7–132.0	133.28	19.85	109.8–158	0.07
NLR	2.87	2.20	0.14–4.48	3.58	3.16	0.90–11.46	4.75	5	1.16–14.6	0.00

**Table 3:** Laboratory biomarkers according to the severity of COVID-19 (year 2021).

Year 2021		Mean	Stdev	Range	Mean	Stdev	Range	Mean	Stdev	Range	*p* Value
Biological Variables *		Mild Form			Moderate Form			Severe Form			
Neutrophils (×10^3^/µL)	COVID-19	2.80	0.97	2.11–3.48	5.29	2.20	2.82–11.44	7.75	3.29	3.50–12.52	0.02
	PCI **	6.66	1.55	4.72–8.40	7.07	3.24	3.24–14.65	7.07	3.24	3.24–14.65	0.14
Ly (×10^3^/µL)	COVID-19	1.12	0.07	1.07–1.17	1.34	0.64	0.48–2.46	2.04	2.66	0.60–9.04	0.69
	PCI	2.24	0.57	1.35–2.84	2.01	0.98	0.6–5.28	1.63	0.72	0.40–3.65	0.06
PLT(×10^3^/µL)	COVID-19	106.50	74.25	54.00–159	234.15	86.38	123.00–355.00	331.33	123.601	72.00–564.00	0.03
	PCI	260.00	80.50	136.00–365	319.20	127.32	127.32–660.00	319.20	127.32	127.32–660.00	0.43
CRP (mg/L)	COVID-19	28.90	28.43	8.80–49	82.22	81.50	14.20–296.00	131.30	89.70	8.00–27.05	0.01
	PCI	8.72	9.63	2.10–28	58.48	93.63	0.6–323.90	50.36	65.65	1.60–232.400	0.00
ESR (mm/1 h) ***	COVID-19	26.00	2.83	24.00–28	37.00	17.81	5.00–70.00	68.11	33.38	15.00–115.00	0.03
	PCI	31.33	18.39	8.00–60	67.96	43.88	8–140.00	68.35	35.84	16–140.00	
LDH (U/L)	COVID-19	164.00	18.38	151.00–177	287.81	133.09	147.00–649.00	554.43	557.56	202.90–1998.40	0.04
	PCI	247.48	105.92	145.00–381	284.12	122.64	129.90–659.00	305.44	130.98	114.20–687.80	0.00
Glucose (mg/dL)	COVID-19	138.00	50.91	102.00–174	129.68	65.48	82.30–318.00	173.80	75.35	83.80–324.00	0.09
	PCI	119.45	31.63	90.20–178.5	125.72	62.87	62.87–349.10	124.21	50.23	66.60–240.30	0.00
NLR	COVID-19	2.50	1.02	1.80–3.25	5.18	5.83	1.29–6.65	6.41	3.48	0.81–10.84	0.23
	PCI	3.11	0.96	1.90–4.57	4.57	3.68	1.18–15.42	7.46	6.78	1.33–10.60	0.04

* Biological variables are presented in Table 2. ** PCI—Post COVID-19 infection. *** ESR—Erythrocyte Sedimentation Rate (mm/1 h).

**Table 4:** Differences in laboratory biomarkers according to the severity of COVID-19/PCI infection (year 2022).

Year 2022		Mean	Stdev	Range	Mean	Stdev	Range	Mean	Stdev	Range	*p* Value
Biological Variables		Mild Form			Moderate Form			Severe Form			
Neutrophils(×10^3^/µL)	COVID-19	4.55	1.16	2.88–5.90	5.67	3.37	2.07–11.28	5.10	1.92	1.92–7.22	0.83
	PCI	4.54	2.22	2.58–9.83	6.83	4.31	1.09–18.91	9.00	4.99	2.25–18.36	0.02
Ly (×10^3^/µL)	COVID-19	1.32	0.54	0.51–2.05	0.91	0.39	0.45–1.49	1.60	1.00	0.62–2.61	0.54
	PCI	1.63	0.76	0.68–3.03	1.69	0.77	0.57–3.16	1.95	1.48	0.56–4.62	0.82
PLT(×10^3^/µL)	COVID-19	234.33	36.97	189–300	226.00	117.02	115–413	182.67	15.82	15.8–200	0.36
	PCI	275.33	77.96	166–368	305.06	90.37	125–499	294.18	98.93	162–438	0.71
CRP (mg/L)	COVID-19	32.20	43.60	1.60–111	22.90	16.20	1.7–44.90	56.90	42.40	14.3–99.10	0.26
	PCI	15.80	29.49	0.6–90.4	33.75	32.21	1.1–120	75.02	111.19	5–381.6	0.01
ESR (mm/1 h)	COVID-19	22.00	15.89	4.00–40	31.71	34.02	7–105.0	23.00	11.53	11.53–35	0.73
	PCI	33.00	34.06	6.00–110	49.26	32.64	1–110	70.20	43.03	15–130	0.04
LDH (U/L)	COVID-19	185.43	35.93	137.7–222.7	252.14	71.31	156–381	282.83	45.18	45.1–324	0.02
	PCI	210.04	45.59	156–312	255.18	89.65	123–478	289.78	107.38	145.7–489	0.05
Glucose (mg/dL)	COVID-19	119.33	24.76	96–151	136.44	43.17	82.1–194	150.50	58.65	58.6–218	0.38
	PCI	120.08	70.61	89.4–307.7	106.35	33.51	63.1–245	110.74	43.59	76.2–226.8	0.82
NLR	COVID-19	3.99	1.74	1.97–6.28	7.17	6.65	3.42–7.57	5.20	5.58	1.77–11.65	0.23
	PCI	3.52	2.67	0.86–4.73	5.06	5.20	1.03–10.26	9.35	10.11	0.9–12.79	0.20

**Table 5:** Mann–Whitney U test applied to contents of biological parameters in the case of patients with COVID-19/PCI.

Mann–Whitney U Test							
	Rank Sum—COVID-19/2021	Rank Sum—COVID-19/2020	U	Z	*p*-value	Valid N—COVID-19/2021	Valid N—COVID-19/2020
N	973	857	191	3.629	*p* < 0.005	24	36
CRP	1130	700	34	5.998	*p* < 0.005	24	36
Age	903.5	926.5	260.5	2.580	*p* < 0.005	24	36
	**Rank Sum—COVID-19/2020**	**Rank Sum—COVID-19/2022**	**U**	**Z**	***p*-value**	**Valid N—COVID-19/2020**	**Valid N—COVID-19/2022**
N	831	547	165	−2.429	*p* < 0.005	36	16
CRP	745	633	79	−4.134	*p* < 0.005	36	16
ESR	1054.5	323.5	187.5	1.983	*p* < 0.05	36	16
Hospitalization time	1115	263	127	3.182	*p* < 0.005	36	16
Age	761.5	616.5	95.5	−3.807	*p* < 0.005	36	16
	**Rank Sum—COVID-19/2021**	**Rank Sum—PCI/2022**	**U**	**Z**	***p*-value**	**Valid N—COVID-19/2021**	**Valid N—PCI/2022**
CRP	1230.5	1619.5	293.5	3.612	*p* < 0.005	24	51
LDH	1086.5	1763.5	437.5	1.976	*p* < 0.05	24	51
Glucose	1136.5	1713.5	387.5	2.544	*p* < 0.05	24	51
Hospitalization time	1299.5	1550.5	224.5	4.395	*p* < 0.0001	24	51
	**Rank Sum—COVID-19/2021**	**Rank Sum—PCI/2021**	**U**	**Z**	***p*-value**	**Valid N—COVID-19/2021**	**Valid N—PCI/2021**
N	732.5	2042.5	432.5	−1.928	*p* < 0.05	24	50
Ly	642	2133	342	−2.973	*p* < 0.005	24	50
LDH	1205.5	1569.5	294.5	3.522	*p* < 0.0005	24	50
	**Rank Sum—COVID-19/2022**	**Rank Sum—PCI/2022**	**U**	**Z**	***p*-value**	**Valid N—COVID-19/2022**	**Valid N—PCI/2022**
Ly	396.5	1881.5	260.5	−2.162	*p* < 0.05	16	51
PLT	341	1937	205	−2.978	*p* < 0.005	16	51
ESR	380	1831	244	−2.327	*p* < 0.05	16	50
Glucose	731	1547	221	2.743	*p* < 0.005	16	51

## Data Availability

Data are contained within the article.

## Supplementary Materials

The following supporting information can be downloaded at: https://www.mdpi.com/article/10.3390/jpm14020171/s1, Table S1: Concentration mean and confident interval (CI) in case of biological parameters for patients with COVID-19 and Post Covid infection.
