# Supplementary material for: Laboratory Diagnostics Accuracy for COVID-19 versus Post-COVID-19 Syndrome in Lung Disease Patients with Multimorbidity
Source: J Pers Med. 2024 Jan 31;14(2):171. doi: 10.3390/jpm14020171 (PMC10890354; doi:10.3390/jpm14020171)
Supplement: Supplementary file 1 [file jpm-14-00171-s001.zip › jpm-2829788-supplementary.pdf]

Table S1. Concentration mean and confident interval (CI) in case of biological parameters for patients with COVID-19 and Post Covid infection

	N_COVID19 (95% CI)	N_PCI (95% CI)	Ly_COVID19 (95% CI)	Ly_PCI (95% CI)	PLT_COVID19 (95% CI)	PLT_PCI (95% CI)	CRP_COVID19 (95% CI)	CRP_PCI (95% CI)	LDH_COVID19 (95% CI)	LDH_PCI (95% CI)
**2021**										
Hypertension	5.70 (2.82 - 11.44)	5.68 (1.55 - 10.60)	1.86 (0.48 - 9.04)	1.54 (0.40 - 2.43)	251.00 (54.00 - 378.00)	283.80 (112.00 - 397.00)	138.25 (88.00 - 296.00)	100.99 (68.60 - 161.90)	294.43 (147.00 - 649.00)	276.30 (150.00 - 430.00)
Obesity	6.46 (6.02 - 6.90)	8.03 (2.97 - 14.65)	0.79 (0.70 - 0.87)	1.84 (1.14 - 2.30)	175.00 (172.00 - 178.00)	289.00 (108.00 - 507.00)	141.19 (90.00 - 193.38)	89.53 (2.10 - 323.90)	1998.40 (1998.40 - 1998.40)	271.35 (129.90 - 490.00)
Diabetes mellitus	5.43 (3.48 - 10.53)	4.09 (3.08 - 5.09)	1.36 (1.06 - 2.08)	1.93 (1.47 - 2.39)	269.67 (54.00 - 378.00)	267.00 (145.00 - 389.00)	78.47 (88.00 - 186.50)	153.20 (73.90 - 232.50)	291.82 (151.00 - 583.90)	385.00 (140.00 - 530.00)
Ashma	4.21 (4.21 - 4.21)	6.31 (4.72 - 7.26)	1.41 (1.13 - 1.69)	2.69 (2.48 - 2.84)	249.00 (160.00 - 338.00)	264.33 (230.00 - 327.00)	73.00 (25.40 - 120.50)	5.27 (2.10 - 7.30)	219.50 (189.00 - 250.00)	248.47 (171.10 - 380.60)
Cardiac diseases	7.70 (3.48 - 11.92)	5.41 (1.55 - 9.26)	1.09 (1.07 - 1.10)	0.93 (0.40 - 1.46)	309.00 (54.00 - 564.00)	305.50 (235.00 - 376.00)	84.00 (80.00 - 88.00)	76.85 (68.60 - 85.10)	264.95 (151.00 - 378.90)	365.00 (150.00 - 480.00)
Hypothyroidism		3.77 (1.55 - 9.42)		1.13 (0.56 - 1.99)		257.75 (164.00 - 397.00)		92.18 (85.10 - 113.00)		279.50 (170.00 - 488.00)
COPD		9.03 (3.68 - 17.91)		1.08 (0.36 - 1.45)		369.00 (162.00 - 441.00)		80.31 (1.10 - 391.60)		421.14 (156.00 - 589.00)
Pleurisy		7.97 (3.15 - 9.11)		1.62 (0.57 - 2.91)		453.90 (140.00 - 599.00)		42.38 (7.20 - 120.90)		359.21 (217.00 - 478.00)
NBP		14.44 (3.62 - 20.36)		1.96 (0.56 - 4.12)		564.00 (162.00 - 699.00)		109.13 (4.10 - 381.60)		416.00 (169.00 - 689.00)
Chronic renal diseases	3.73 (3.48 - 4.21)		1.12 (0.60 - 1.69)		217.67 (54.00 - 338.00)		67.10 (8.80 - 167.20)		216.60 (151.00 - 282.20)	
**2022**										
Hypertension	4.77 (2.07 - 11.28)	6.01 (1.09 - 18.36)	1.05 (0.35 - 2.05)	1.95 (0.56 - 4.62)	218.58 (115.00 - 413.00)	292.14 (125.00 - 499.00)	22.40 (1.60 - 76.80)	45.48 (1.10 - 381.60)	239.17 (145.20 - 381.20)	266.80 (156.00 - 489.00)
Obesity	3.92 (2.26 - 4.84)	7.69 (2.25 - 18.36)	1.37 (0.35 - 2.61)	1.64 (0.56 - 3.39)	193.50 (152.00 - 300.00)	310.17 (162.00 - 438.00)	40.90 (9.20 - 76.80)	78.12 (1.10 - 381.60)	267.93 (137.70 - 324.00)	251.70 (156.00 - 489.00)
Diabetes mellitus	4.43 (2.26 - 7.13)	7.57 (1.09 - 18.91)	0.92 (0.35 - 1.49)	1.65 (0.56 - 3.94)	223.50 (152.00 - 413.00)	301.36 (162.00 - 438.00)	40.10 (18.10 - 76.80)	65.82 (1.10 - 381.60)	258.95 (202.00 - 323.00)	277.56 (156.00 - 489.00)
Asthma		5.40 (2.31 - 7.26)		1.71 (1.03 - 2.74)		274.00 (162.00 - 333.00)		24.13 (6.40 - 38.40)		229.17 (193.70 - 255.00)
Cardiac diseases	5.49 (2.07 - 11.28)	9.02 (2.31 - 18.91)	0.81 (0.35 - 1.57)	1.37 (0.56 - 2.73)	205.11 (142.00 - 367.00)	277.91 (140.00 - 499.00)	39.70 (1.60 - 111.00)	71.14 (2.00 - 381.60)	251.49 (156.00 - 381.20)	293.03 (156.00 - 489.00)
COPD	3.97 (2.07 - 4.97)	11.03 (3.68 - 18.91)	1.60 (0.45 - 2.61)	1.08 (0.56 - 1.80)	197.40 (142.00 - 263.00)	269.00 (162.00 - 341.00)	24.90 (12.00 - 57.20)	84.61 (1.10 - 381.60)	273.40 (156.80 - 381.20)	321.14 (156.00 - 489.00)
Hypotiroidism		4.77 (1.65 - 8.42)		1.15 (0.40 - 1.92)		357.75 (164.00 - 497.00)		62.18 (45.10 - 213.00)		284.00 (170.00 - 488.00)
Pleurisy		5.97 (3.55 - 9.11)		1.52 (0.57 - 2.71)		293.90 (140.00 - 499.00)		42.38 (7.20 - 120.90)		289.21 (217.00 - 378.00)
Tuberculosis		12.44 (2.62 - 18.36)		1.66 (0.56 - 3.12)		364.00 (162.00 - 499.00)		109.13 (4.10 - 381.60)		316.00 (169.00 - 489.00)

N=neutrophils; Ly=lymphocytes; PLT=platelets; CRP=C-reactive protein; LDH=lactate-dehydrogenase
